# Synthesis of Nm-PHB (nanomelanin-polyhydroxy butyrate) nanocomposite film and its protective effect against biofilm-forming multi drug resistant *Staphylococcus aureus*

**DOI:** 10.1038/s41598-017-08816-y

**Published:** 2017-08-22

**Authors:** George Seghal Kiran, Stephen A. Jackson, Sethu Priyadharsini, Alan D. W. Dobson, Joseph Selvin

**Affiliations:** 1 Department of Food Science and Technology, Pondicherry University, Puducherry, 605014 India; 2 School of Microbiology, University College Cork, Cork, Ireland; 3 Environmental Research Institute, University College Cork, Cork, Ireland; 4 Department of Microbiology, School of Life Sciences, Pondicherry University, Puducherry, 605014 India

## Abstract

Melanin is a dark brown ubiquitous photosynthetic pigment which have many varied and ever expanding applications in fabrication of radio-protective materials, food packaging, cosmetics and in medicine. In this study, melanin production in a *Pseudomonas* sp. which was isolated from the marine sponge *Tetyrina citirna* was optimized employing one-factor at a time experiments and characterized for chemical nature and stability. Following sonication nucleated nanomelanin (Nm) particles were formed and evaluated for antibacterial and antioxidant properties. Nanocomposite film was fabricated using combinations (% w/v) of polyhydroxy butyrate-nanomelanin (PHB:Nm) blended with 1% glycerol. The Nm was found to be spherical in shape with a diameter of 100–140 nm and showed strong antimicrobial activity against both Gram positive and Gram negative bacteria. The Nm-PHB nanocomposite film was homogeneous, smooth, without any cracks, and flexible. XRD and DSC data indicated that the film was crystalline in nature, and was thermostable up to 281.87 °C. This study represents the first report on the synthesis of Nm and fabrication of Nm-PHB nanocomposite film which show strong protective effect against multidrug resistant *Staphyloccoccus aureus*. Thus this Nm-PHB nanocomposite film may find utility as packaging material for food products by protecting the food products from oxidation and bacterial contamination.

## Introduction

Melanins are biologically important ubiquitous pigments that are found in all kingdoms of life. They typically provide protection from environmental stress, such as against ultraviolet rays from the sun and energy transduction. They are complex and diverse heteropolymeric dark black or brown pigments formed by oxidative polymerization of phenolic and/or indolic compounds or by polyketide synthase pathways. Tyrosinase enzymes are involved in the best known melanin biosynthetic pathway, by producing phaeomelanins (red) or eumelanins through the oxidation of tyrosine to l-3,4-dihydroxyphenylalanine (l-DOPA) and dopaquinone^1^. Melanin production and tyrosinase activity in soil bacteria such as *Azospirillum* and *Rhizobium* sp. is known to play a role in their symbiotic relationship with plants^2^. However, melanin production is also important in the pathogenicity of several bacterial and fungal species. It can directly affect the immune response of the host towards infection by increasing the resistance of the pathogen to the host’s antimicrobial mechanisms. Subsequently, melanin biosynthetic pathways and melanin itself are both potential targets for the discovery of antimicrobial drugs. Melanins are negatively charged, hydrophobic, amorphous and high molecular weight compounds that are resistant to chemical degradation by acids and by oxidizing agents^3, 4^. Synthetic melanins can be prepared by chemical oxidation of dopamine or by enzymatic oxidation^5^. Synthetic melanin particles are not uniform in size and are insoluble in water, thus size-controlled synthesis methods are not available for synthetic melanin particles^6^.

Melanins have many and varied commercial applications, including their use in the preparation of cosmetics as a sun protection factor (SPF), where they function by absorbing radiation and preventing photo induced damage to the skin^7^. By protecting against cellular damage induced by ultraviolet (UV) B it also helps protect from certain skin diseases^8–10^. They also find application as antioxidants, as antitumor and anti-inflammatory agents^11^, and as immune stimulating agents^12^. They have also been used in semiconductor and in bio- electronics applications as well as in polarized sunglass lenses, paints and varnishes. Since melanins are well-known to protects cells from ionizing radiation, melanin incorporated films can be used as radio-protective materials. However, thin melanin films are typically quite fragile, and are often stabilized with plasticisers such as glycerol, organic solvents or water to increase their durability^13^. Synthetic melanin film has been prepared by coating with silicon dioxide^14^. High quality melanin thin films have important electronic applications that can be prepared by the oxidation of dopamine, but the available process is time consuming and typically generates an uneven granular morphology^15–17^.

The main intention of food packaging materials is to provide protection from oxidation, microbial spoilage, radiation and oxidative processes. To replace synthetic plastics in food packaging, new sources of edible / biodegradable food packing materials are being developed based on various bioplastics and microbial products^18^. PHB (polyhydroxy butyrate) has gained increased attention due to its wide spread use in medical, food packaging and anti-adhesive applications. While PHB has high thermal stability and crystallinity, processing it into thin flexible film is a challenging process^19^. However using a combinatorial approach such as heat treatment, co-polymerization^20, 21^ blending^22, 23^ and the addition of plasticizers^24, 25^, PHB can be made into flexible films with high thermal and mechanical properties. A polymer such as PHB has a crystalline phase, which provides good mechanical, and permeation properties, and thus constitutes a suitable and safe packaging material. PHB blended with PLA (poly lactic acid) has been used in a variety of food packaging applications, because of its high degree of biodegradability^26^. Nanoscale systems have also gained increased popularity for efficient drug and chemotherapy delivery, which exhibit reduced side effects^27^.

Bacteria from marine sources such as *Pseudomonas stutzeri* isolated from the seaweed *Hypnea musciformis* and marine sediments has previously been reported as a melanin producer^28, 29^. Marine sponges are known to be the source of complex microbial communities including bacteria, fungi and archaea of ecological and biotechnological importance. Marine sponge associated microorganisms can constitute up to 40% of the overall sponge biomass and are known to produce novel antimicrobial agents, bioactive compounds, antifouling agents and numerous enzymes of industrial interest^30–32^. The marine sponge associated actinobacterium *Nocardiopsis alba* MSA10 has also previously been exploited for melanin production^33^. We report here on the production of melanin from a deep sea sponge associated *Pseudomonas* strain, optimization of melanin production, synthesis of nanomelanin (Nm) and the fabrication of Nm-PHB nanocomposite film, prepared by blending PHB with melanin. The aim was to produce a film with increased durability, with the potential to protect against microbial colonization and biofilm formation. To this end, the Nm-PHB nanocomposite film was characterized for thermal stability, together with structural and morphological properties, along with its anti-infective effect against multiple-drug resistant *Staphylococcus aureus* (MDRSA).

## Results

### Identification of Melanin producer

**Figure 1:**
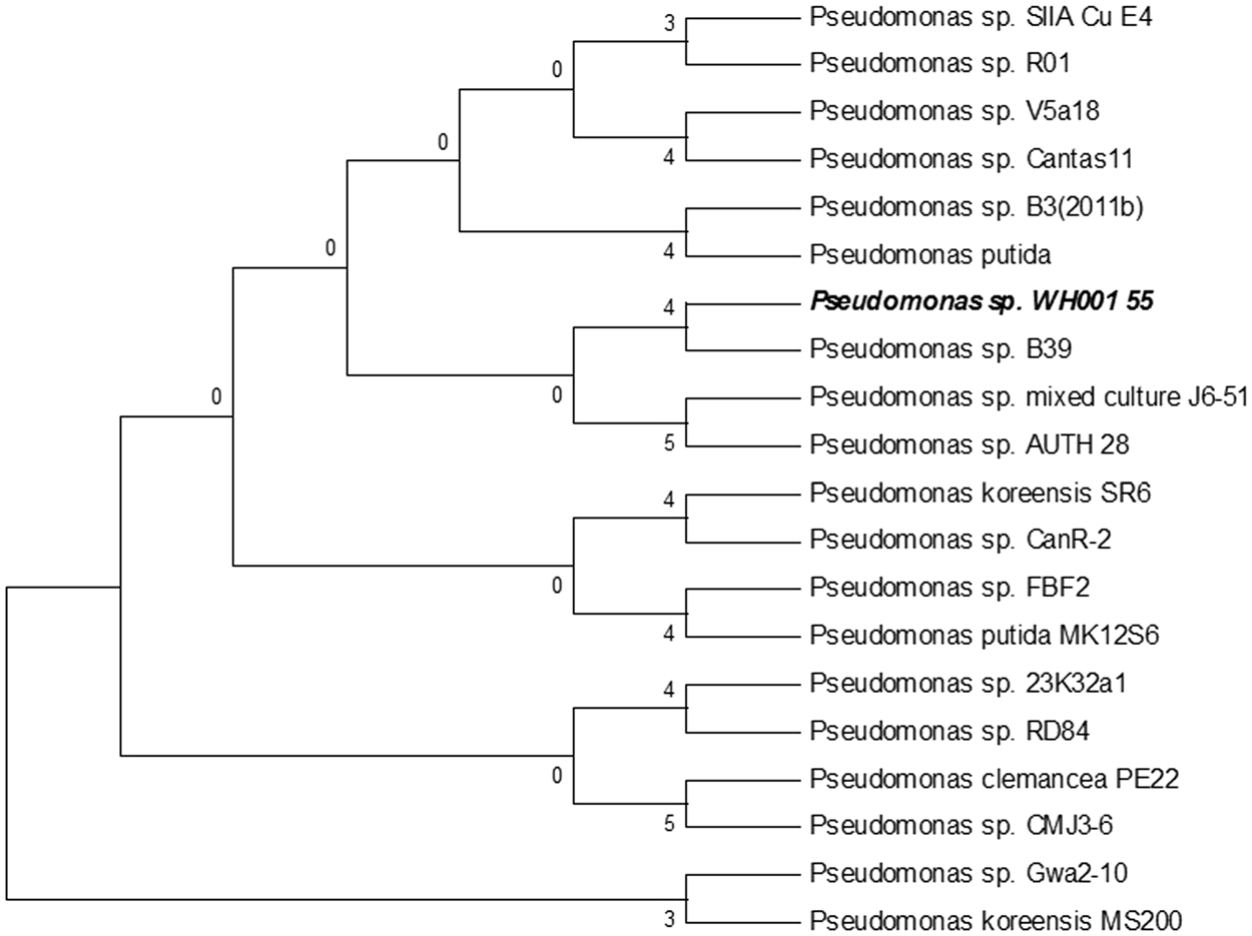
The evolutionary tree was inferred using the Minimum Evolution (ME) method. The bootstrap consensus tree inferred from 1000 replicates was taken to represent the evolutionary history of the taxa analyzed. The phylogenic tree was constructed using the ME algorithm in MEGA version 7.0. The isolate showed 97% similarity with members of the *Pseudomonas* genera.

A total of 66 distinct bacteria were isolated from the marine sponge *Tethya citrina* on SYP-SW agar plates. All the isolates were screened for melanin production, with isolates WH001 55 and WH001 82 being identified as the best melanin producers. The strain WH001 55 was selected for further study based on its ability to produce higher levels of melanin of 6.2 mg/ml as opposed to 3.2 mg/ml which was produced by WH001 82. The isolate WH001 55 was identified as a rod shaped Gram-negative bacterium, which produced a light yellow colour when grown on SYP-SW agar. Based on the morphological and phylogenetic analysis (ME algorithm) and taxonomic affiliation (RDP-II), the isolate WH001 55 was identified as a *Pseudomonas* sp. (Figure 1). The 16 S rRNA sequence was deposited in Genbank with an accession number KX390669. The strain WH001 55 also produced a yellow colour with zone formation on tyrosine agar plates occurring after 48 h of growth, with colour formation progressively increasing and turning a dark brown after 144 h.

### Production of melanin

**Figure 2:**
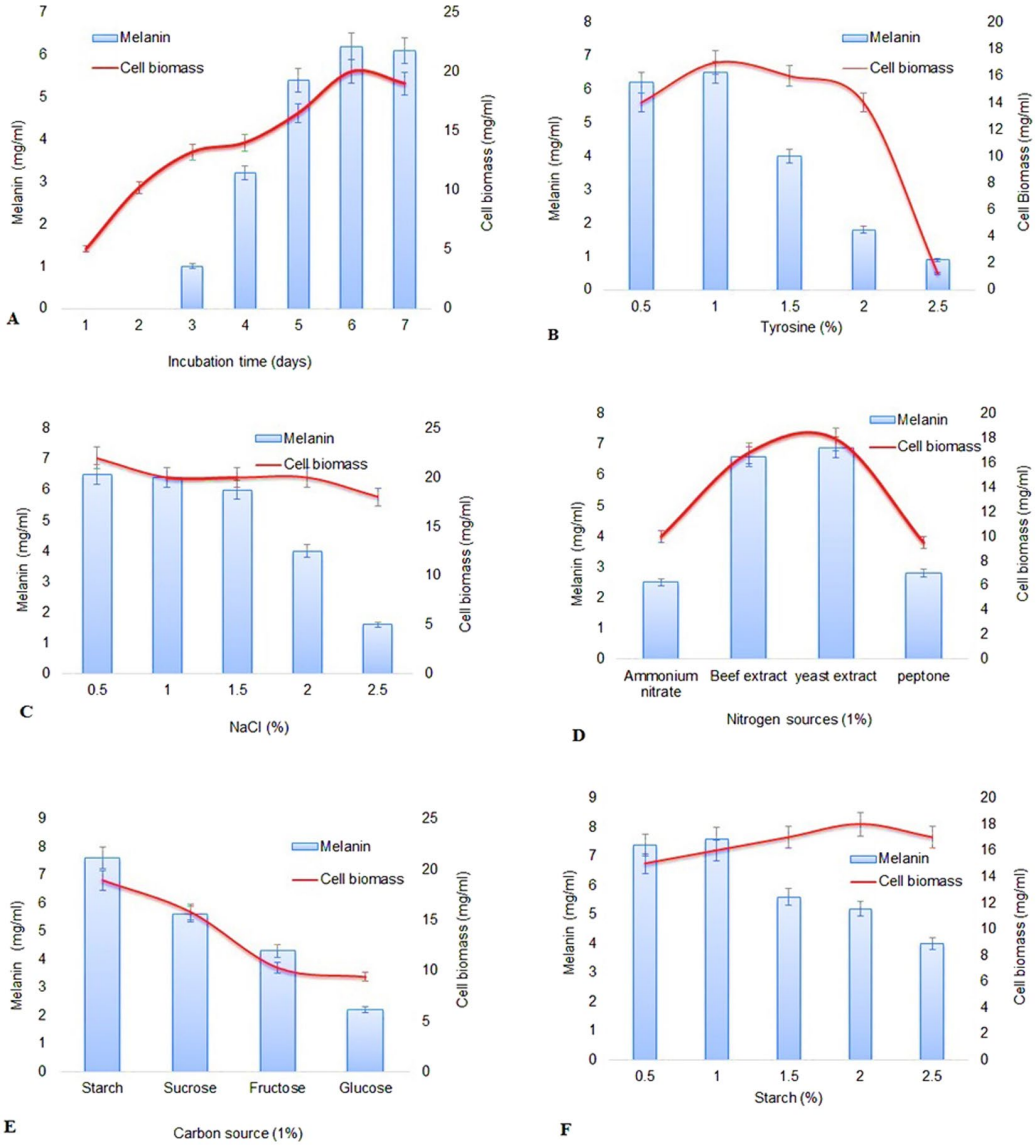
Effect of nutritional factors on cell biomass and melanin production. (**A** and **B**) Show optimized production of melanin at various incubation times and the effect of tyrosine supplementation in the production media. (**C**–**F**) Show the effect of supplementation with NaCl and nutritional sources such as nitrogen and carbon.

The effect of various nutritional factors and incubation time, affecting cell biomass and melanin production in WH001 55 was assessed (Fig. 2). Maximum production of biomass (20 mg/ml) and melanin yield (6.2 mg/ml) was observed on the 6th day of incubation, with melanin production being concomitant with increased cell biomass (Fig. 2A). The requirement of tyrosine for enhanced melanin production was evaluated, and supplementation of the production medium with 1% tyrosine was found to yield maximum biomass (17 mg/ml) and melanin (6.5 mg/ml) (Fig. 2B). The effect of nutritional factors required for enhanced melanin production was subsequently assessed in production media supplemented with 1% tyrosine (Fig. 2C–F). Being a marine isolate, biomass levels in strain WH001 55 were consistently high in the media supplemented with 0.5–1.5% NaCl and decreased between 2% and 2.5%. Concomitant changes in melanin production were also observed, with maximum melanin production being observed in the production media supplemented with 0.5% NaCl (6.5 mg/ml), (Fig. 2C). Supplementation of the medium with various nitrogen sources tested did not have a marked effect on the cell biomass levels, however melanin yield was slightly increased with supplementation of nitrogen sources such as yeast extracts, with 1% yeast extract yielding 6.9 mg/ml melanin in the production media (Fig. 2D). Of the carbon sources tested, supplementation with 1% starch resulted in enhanced production of melanin (7.6 mg/ml) and cell biomass (18.9 mg/ml), (Fig. 2E). While higher levels of starch supplementation in the production media resulted in higher cell biomass levels, 1% starch was found to be the optimal level for melanin production in the medium (Fig. 2F). This optimized media was used for scale-up production of melanin in shake-flask cultures and subsequently used in the extraction and preparation of Nm and nanocomposite films.

### Stability and characteristics of melanin

**Figure 3:**
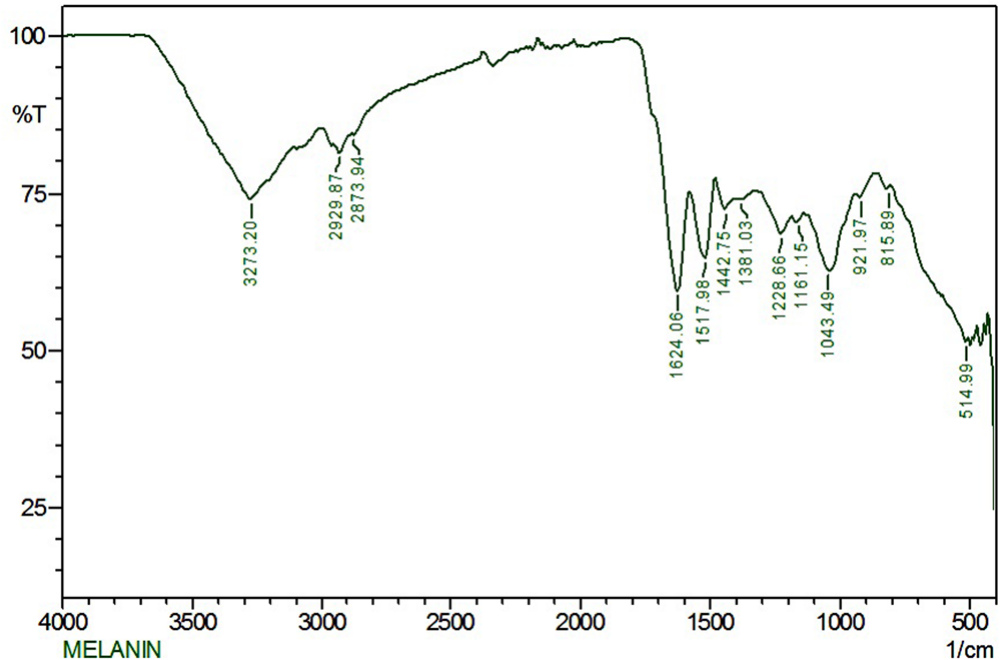
FTIR spectra of melanin showing a broad absorption spectra at 3273 cm^−1^ which may be due to characteristic O-H stretching or N-H stretching vibrations of the carboxylic acid, and phenolic groups in melanin.

A strong peak characteristic of melanin was observed at 220 nm in the UV spectrum. Melanin was exposed to various temperatures ranging from between 40 and 100 °C and was found to be stable to both UV irradiation and high temperatures. The extracted melanin was readily soluble in hexane, water (pH above 7.0) and DMSO. Melanin was insoluble in organic solvents including ether, ethyl acetate, methanol and ethanol. TLC analysis showed a violet coloured spot corresponding to an Rf value of 0.68 related to the pigment melanin. Subsequent purification of this TLC spot following DEAE-Cellulose chromatography resulted in a melanin fraction which upon FT-IR analysis, showed a broad absorption at 3273 cm^−1^ (Fig. 3). This appears to indicate the presence of associated or polymeric - OH groups or N-H stretching vibrations of the carboxylic acid, and phenolic groups of melanin; while smaller bands at 2929 cm^−1^ and 2873 cm^−1^ and may be a result of stretching vibrations of the aliphatic C-H groups. The characteristic strong band at 1625 cm^−1^ can be attributed to vibrations of aromatic ring C = C of amide I, C = O and/or of COO- groups, while bands at ~1400 to 1500 cm^−1^ may be due to aliphatic C-H groups.

### Characteristics of Nm

**Figure 4:**
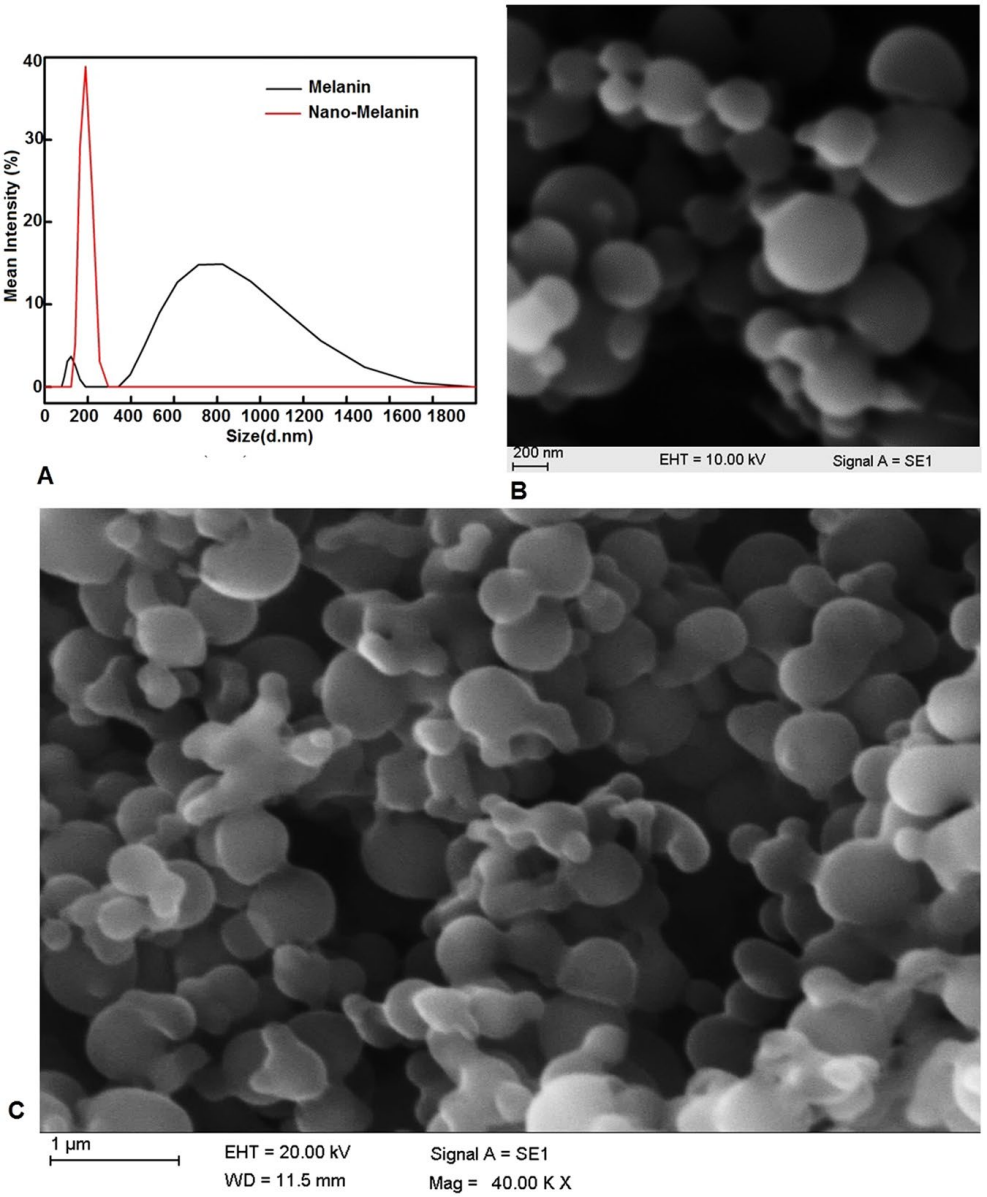
(**A**) peaks obtained for nanomelanin (black) and melanin (red) in the particle size anaylzer. (**B**–**C**) shows the SEM images of the nanomelanin.

The particle size was determined for the control melanin produced by *Pseudomonas* sp. WH00155 and for the nanomelanin. The size distribution of particles of the control melanin were in the range of 122.4 nm (with a smaller intensity of 11.4% and width of 19.87) and 820.4 nm (with a larger intensity of 88.6% and width 261.5) with a mean diameter of 519.6. However, the Nm showed a single peak with particle size of 189.3 nm with 100% intensity with a mean of 208.7 nm (Fig. 4A). The diameter of the Nm particles was measured using an SEM micrograph, and these spherical shaped particles had a diameter of 100–140 nm (Fig. 4B,C).

The free radical scavenging effect of Nm was assessed using the DPPH assay, with different concentrations of Nm ranging from between 20 and 120 µg ml^−1^ being employed. The free radical scavenging activity increased at higher Nm concentrations. Nm exhibited a high free radical scavenging activity of 67.8% comparable with ascorbic acid (positive control) which showed a scavenging activity of 64.08% (Supplementary Figure S1A). The antioxidant activity increased with increased Nm concentrations and with an increase in reaction time (Supplementary Figure S1B). The antimicrobial properties of melanin and Nm was then assessed against a range of bacterial strains (Supplementary Figure S1C). Zones of inhibition were observed with (a) *Staphylococcus aureus*, (b) *Escherichia coli*, (c) *Pseudomonas aeruginosa a*nd (d) *Bacillus subtilus* sp. after 24 h of incubation, with Nm displaying slightly stronger antibacterial activity than the melanin. In the microtitre plate assay, Nm (30 µg/ml) showed effective inhibition of *S*. *aureus* followed by *E*. *coli, P*. *aeruginosa* and *B*. *subtilis* (Supplementary Figure S1D), whereas melanin (80 µg/ml) showed a medium level of antibacterial activity against *S*. *aureus* followed by *E*. *coli, P*. *aeruginosa* and *B*. *subtilis*. Based on the MBC:MIC ratio, the mode of action of Nm was classified as “bactericidal” (Supplementary Tables S1 and S2). The antimicrobial activity was effected on both Gram negative and Gram positive bacteria and therefore Nm was grouped under broad-spectrum antibacterial compound.

### Characteristics of Nm-PHB film

**Figure 5:**
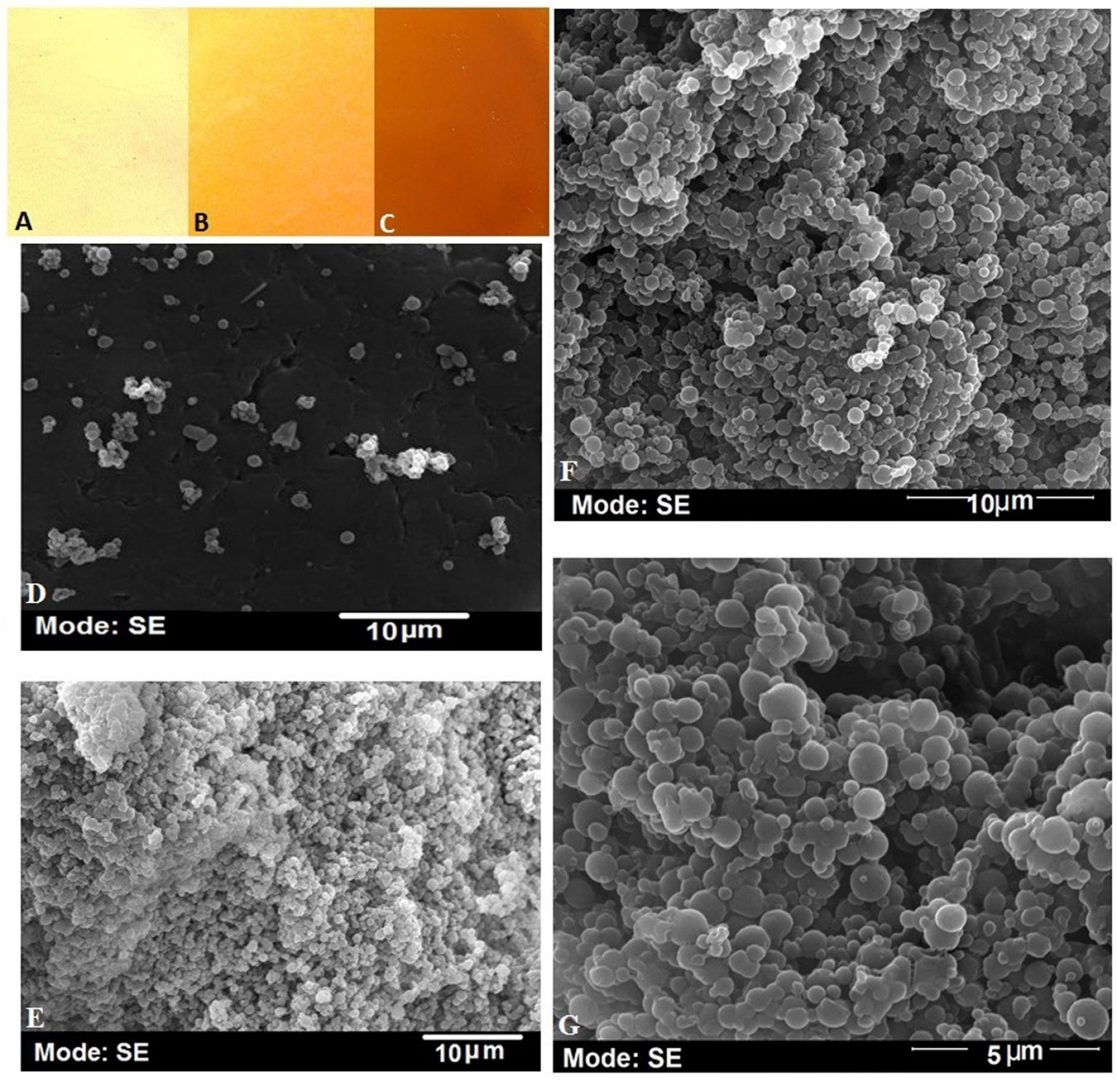
Visual appearance of the film (**A**) film prepared using increased PHB 2% and 1% each of Nm (**B**) film made using equal proportionate of Nm, PHB and glycerol and PHB (**C**) film casted using increased concentration of Nm 2% and 1% each of PHB and glycerol. SEM Imaging of films**-**(**D**) corresponds to the image of film using increased concentration of Nm with equal amount of PHB and glycerol of 1%. (**E**) Shows the film prepared using increased concentration of PHB (2%) with equal amount of Nm and glycerol of 1%. (**F-G**) The film formed using equal proportion of Nm, PHB and glycerol (1:1:1).

The moisture content of the Nm-PHB films dried overnight at 105 °C was 15.24 ± 0.253 (Nm: PHB: glycerol 1:1:1% w/v), 16.01 ± 0.423 (Nm: glycerol: PHB 2:1:1% w/v) and 18.20 ± 0.145 (Nm: glycerol: PHB 1:2:1% w/v) respectively. Visual observation of the films revealed characteristic features as shown in Fig. 5A,B and C. The equal proportion blend (PHB:glycerol:Nm 1:1:1% w/v) displayed a smooth surface appearance and was flexible, whereas the increased Nm concentration of 2% in the blend imparted a brown colour on the film and was more brittle. The film prepared using a blend of Nm:PHB:glycerol (1:2:1% w/v) displayed a rough surface together with a hard texture. SEM image analysis showed a smooth surface with minute cracks in the nanocomposite film prepared with a composition of Nm:PHB:glycerol (2:1:1% w/v) (Fig. 5D), whereas the film prepared using increased PHB, Nm:PHB:glycerol (1:2:1% w/v) showed roughness, highly compact and agglomerated structures (Fig. 5E). This may be due to the high crystalline nature of PHB. The nanocomposite film prepared with equal proportions of PHB: glycerol: Nm (1:1:1% w/v) under different magnifications such as 10  µm and 5 µm revealed a smooth surface morphology. The roughness was reduced and flexibility was improved in the equal proportion nanocomposite film (Fig. 5F and G). Thus the Nm appeared to impart a smooth, homogenous and flexible nature to the nanocomposite film prepared using an equal proportion of the components. The analysis also revealed that glycerol was an effective plasticizer blended in the Nm-PHB film.

### XRD and AFM analysis of nanocomposite film

**Figure 6:**
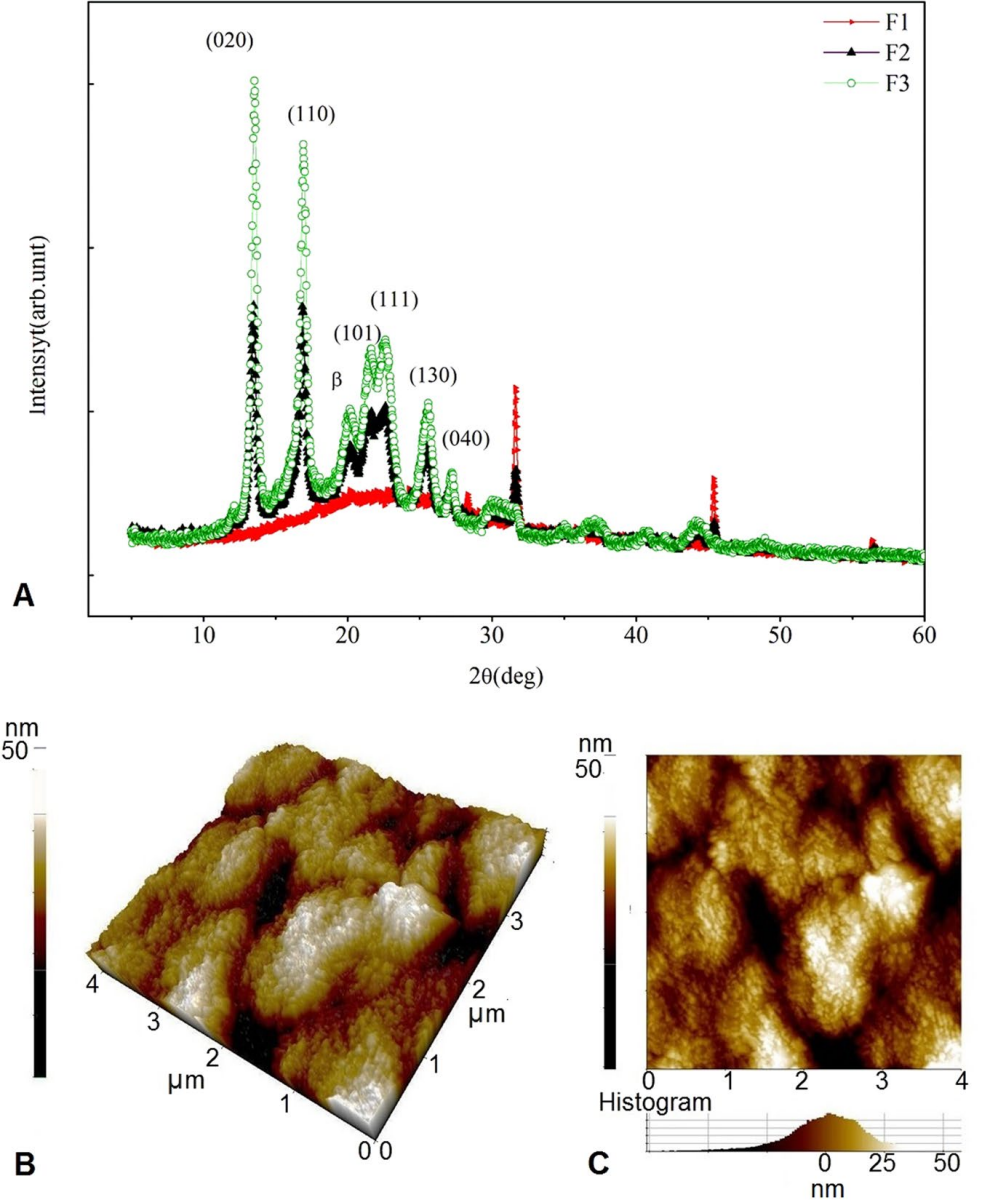
(**A**) X-ray diffractograms of the film. The peaks represented in green (F1) are for the film prepared using increased concentration of PHB, Nm and glycerol blend (2:1:1) with high intensity peaks, indicating the crystallinity of the film. Peaks shown in black (F2) correspond to the film prepared using equal proportion of the components Nm: PHB: glycerol (1:1:1) and displayed a similar diffraction pattern as the peak obtained with the increased concentration of PHB. Peaks represented in red (F3) are the film prepared using increased melanin: PHB: glycerol (2:1:1) which displayed a broader peak in the diffraction pattern of 20.2° to 26.8°. (**B**,**C**) Figure shows the AFM images of the samples scanned in an area of 4μm^2^. AFM topographical images of melanin nanocomposite film prepared with equal proportion of PHB, Nm and glycerol blend (1:1:1). The topographical scale of the images in 3D (**B**) and 2D (**C**) are shown in 50 nm scales. The roughness of the film was found to be 12.4 nm.

The crystallinity of the nanocomposite film was determined by X-ray diffraction (XRD) analysis (Fig. 6A). Peaks corresponding to the nanocomposite film prepared using Nm:PHB:glycerol (1:2:1% w/v) are shown in green (F1), while film prepared using PHB:glycerol:Nm (1:1:1% w/v) is shown in black (F2). In general, both peaks correspond to orthorhombic crystal planes (020), (110), (101) (111) (130) and (040) at 2θ values. Two high intensity sharp peaks were observed at around 13.5°, 16.8° and half width large, while small intensity peaks were detected near to 23.5°, and 24.5°. Other peaks of much smaller intensity were observed at 2θ values of (130) and (040) were 25.6° and 27°. Near to 2θ of 20°, a ß form of crystal was observed. In addition to this, several 2θ values were obtained at 32.6°, 37.6° and 46.3°. The lattice parameter was found to be similar for the film prepared using increased PHB (2%) and equal proportion of the Nm composite film (1:1:1). However, the intensity of the peaks varied with the concentration of PHB in the film. The intensity of the peak was slightly smaller in 1% PHB when compared to film prepared using 2% PHB. In general, melanin is an amorphous polymer whereas PHB is a thermostable crystalline polymer. The broad peaks observed in the XRD analysis depicts an amorphous state while the sharp peaks depict a crystal phase. It can be noted that the film prepared using increased Nm (2%) with 1% each of PHB and glycerol which are represented in red peaks (F3) showed a wider peak with a decrease in intensity ranging from 20.2° to 26.8°, and another peak at 32.6° and 46.3°. The wider peak indicates that increases in Nm concentrations in the blend leads to decreases in the crystallinity. The NanoScope analysis of AFM images (Fig. 6B and C) confirmed nanoscale surface roughness of the film (12.4 nm). The route mean squared (RMS) value is a measure of surface roughness. The AFM imaged confirmed the film was smooth, and without cracks. The AFM data analysis revealed the smooth homogenous surface of the film imparted by Nm.

### Differential Scanning Calorimetry (DSC)

**Figure 7:**
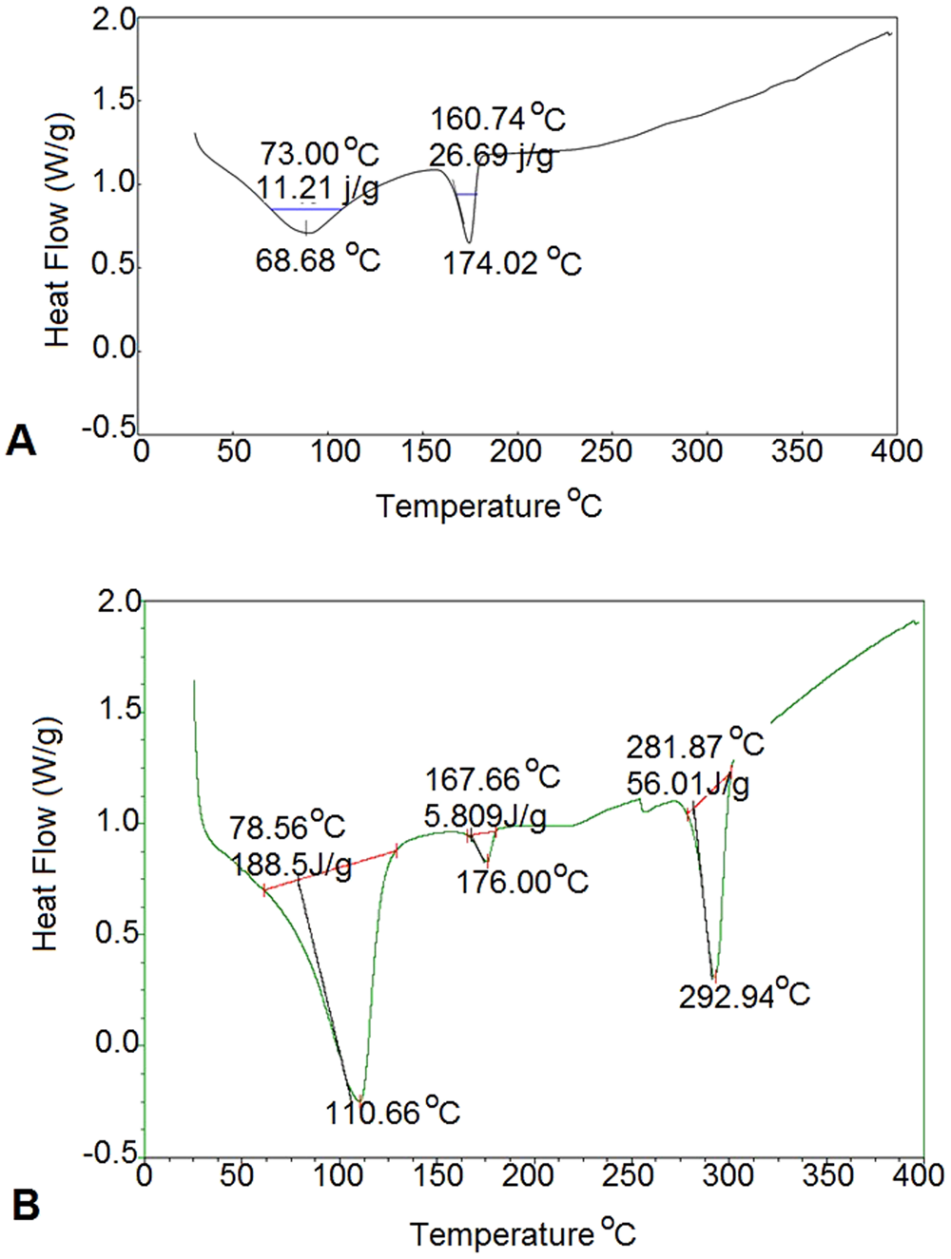
DSC thermograms of melanin (**A**) compared with the DSC of the film prepared using PHB: Nm: Glycerol (**B**). The thermogram showed a highly thermostable peak with a degradation peak at 292.94 °C when compared with the control melanin at 174.02 °C.

The DSC of melanin (control) (Fig. 7A), showed an endothermic peak at 68.68 °C. This may be due to the moisture content present in the sample with an onset temperature of 73.0 °C and 11.21 J/g of latent energy. A second degradation peak appeared in the range of 174.02 °C with an onset temperature of 160.74 °C and 26.69 J/g of latent energy (Fig. 7A). Film blended with PHB, Nm and glycerol (1:1:1 ratio) showed an endothermic peak at a temperature of 110.66 °C which again may be due to the moisture present in the film with an onset temperature of 78.56 °C and a latent energy of 188.5 J/g. A second small peak occurred in the region of 176 °C with an onset temperature of 167.66 °C and latent energy of 5.809 J/g (Fig. 7B). The second peak may belong to the melanin when compared with the control peak in the range of 174.02 °C. A small degradation peak at 265 °C may belong to the glycerol present in the prepared film. The degradation peak that occurred in the range of 292.94 °C with latent energy of 56.01 J/g may belong to PHB, a highly thermostable crystalline polymer. The DSC based experiments revealed that Nm blended with PHB can produce a highly thermo stable crystalline polymer.

### Antibiofilm activity of Nm-PHB composite/film

**Figure 8:**
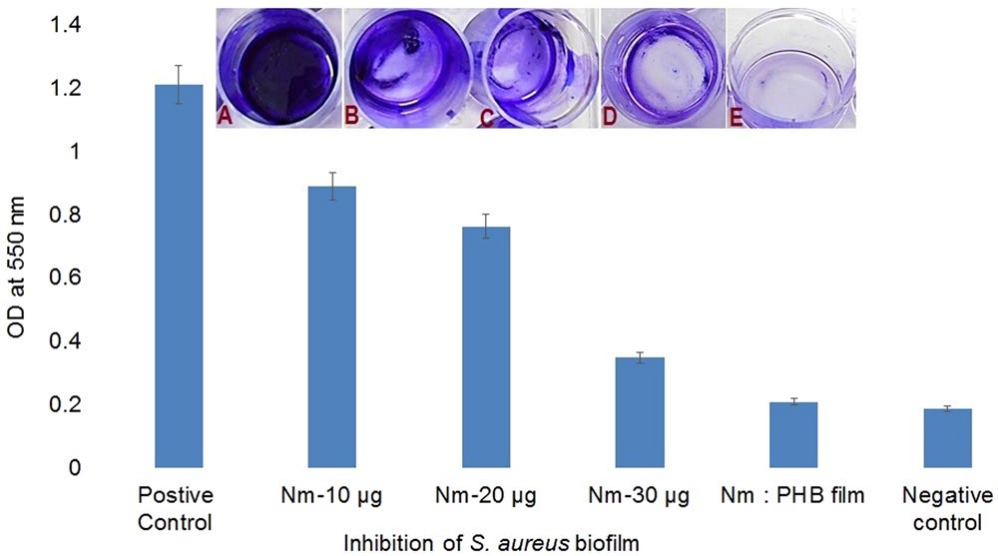
Antibiofilm activity of Nm:PHB composite and Nm was determined quantitatively in a 12-deep well microtiter plate assay. The tested concentrations of Nm (10 and 30 µg/ml) showed antibiofilm activity against *S*. *aureus*. Nm:PHB composite treated wells showed no indication of biofilm formation and were similar to the negative control. The positive control was a biofilm formed by *S*. *aureus*. Inset images are showing top-down view of well plate assay. (**A**) Control biofilm of *S*. *aureus*. (**B**–**D**) Wells coated with Nm 10 (**B**), 20 (**C**) and 30 µg/ml (**D**). (**E**) Wells coated with Nm:PHB composite.

**Figure 9:**
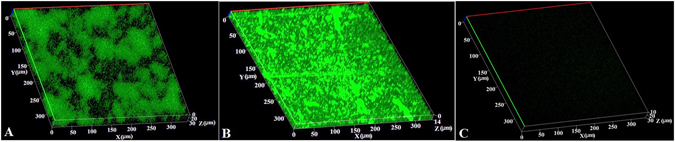
CLSM images showed antibiofilm activity of the Nm-PHB film. Control surfaces such as glass slide (**A**) and µ slide (**B**) shows 100% coverage of biofilm formed by MDR *S*. *aureus*. Image C is showing the antibiofilm effect of Nm-PHB film, which showed no indication of biofilm formed by *S*. *aureus*.

The effect of Nm:PHB composite on biofilm formation was determined quantitatively in a microtiter plate assay (Fig. 8). Nm at varying concentration between 10 and 30 µg/ml showed antibiofilm activity against *S*. *aureus*. The 30 µg/ml Nm treated wells showed a 71% inhibition of the *S*. *aureus* biofilm. A complete inhibition of *S*. *aureus* biofilm was observed in the wells treated with Nm:PHB composite. These wells showed no indication of biofilm formation and were similar to the negative control. The antibiofilm activity was confirmed using confocal laser scanning microscope (CLSM) images with a glass slide / film surface biofilm assay (Fig. 8). The MDR *S*. *aureus* strain was allowed to form a biofilm on the control surfaces include a glass slide and µ slide (ibidi polymer made of high quality plastic) and on the test surface (Nm-PHB film). The control surfaces showed 100% coverage of a thick dense, clump of MDR *S*. *aureus* biofilm (Fig. 9A and B). The Nm-PHB film effectively inhibited the formation of a MDR *S*. *aureus* biofilm, as shown in a CLSM image (Fig. 9C). The Nm-PHB film resulted in complete protection against bacterial colonization, as the CLSM image showed no indication of biofilm formation on the surface of the film.

## Discussion

Marine sponges play host to diverse microbial communities, which can constitute up to 35% of the sponge biomass. These sponge associated microorganisms are known to produce a wide range of secondary metabolites which are thought to provide protection to the sponge from predictors and epibionts^34^. Marine sponges associated microorganisms are also known to produce a wide variety of biotechnologically important enzymes which can be used in the production of pesticides, herbicides and pharmaceuticals^35^. Many of these enzymes possess unique biochemically properties; given that they are functional in bacteria who inhabit these sponges and in doing so are exposed to extremes in temperature, salinity and pressure^36^.

With this in mind, we targeted bacteria from the marine sponge *T*. *citrina* and screened these isolates for melanin pigment production. From these we focused on the isolate *Pseudomonas* sp. WH001 55 which proved the best producer with 6.2 g/l melanin being produced following 144 h of growth. By varying carbon and nitrogen sources, melanin production in *Pseudomonas* sp. WH001-55 was further increased to 7.6 g/l in the production media, with the addition of starch resulting in optimal yields with production flasks turning black after 56 h of incubation due to the high levels of melanin. The levels of melanin produced by *Pseudomonas* sp. WH001-55 were higher than those previously reported for other strains such as *N*. *alba* (3.7 g/l), *Klebsiella* sp. (0.540 g/l), *Frankia* Cel5 (0.180 g/l) and *A*. *fumigatus* AFGRD105 (20 mg/l)^10, 33, 37, 38^. Similar peaks were obtained following FTIR analysis when compared with those from other melanin producing strains and with a melanin standard^39^ and were in the range of 3300 cm^−1^ to 514 cm^−1^. In addition the OH phenolic groups were similar to the peaks previously reported for melanin and were in the range of 1400−1300 cm^−19^.

In this study, we demonstrated that the antibacterial effect of melanin produced by WH001 55 was substantially improved in the nanomelanin preparation. The nanomelanin that were synthesized displayed strong antimicrobial activity against a range of pathogenic bacteria including both Gram positive *S*. *aureus, B*. *subtilis* and Gram negative *E*. *coli*, *P*. *aeruginosa* strains. The results showed antibacterial activity was significantly high in Nm when compared to melanin. The MIC/MBC ratio based mechanism of action revealed Nm was bactericidal. Nanomelanin produced in this study showed that the increased antibacterial effect might be due to the smaller particle size when compared to the melanin. Therefore, nanomelanin may also be useful in anti-infective strategies, potentially in the medical devices area. Melanin has previously been reported as a reducing agent in the synthesis of nanoparticles^33, 40^. Nanoparticle synthesis using current conventional and chemical procedures are typically both quite time consuming and potentially toxic to the environment^41^, therefore safer alternatives are required for more environmentally friendly/greener synthesis of nanoparticles. Our study represents progress in this respect, as the nanomelanin synthesis process we employed did not involve the use of either chemicals or metals.

Visual examination of the Nm-PHB nanocomposite film (1:1:1) revealed that the film surface was homogeneous, smooth, without any cracks, and flexible. The roughness was reduced and the flexibility improved in the equal proportion nanocomposite film. Glycerol was used in the film preparation since it is a colorless, water soluble, odorless component and has wide applicability in the food sector. Incorporation of glycerol into the nanocomposite film appears to have provided flexibility, possibly because of the hydroxyl groups in the glycerol forming inter and intramolecular hydrogen bonding within the polymer. Glycerol has previously been reported to have been successfully employed in film preparations, as a flexible material that enables the packaging process^42^. Glycerol is a hygroscopic molecule that is grouped as a “natural polyol hydrophilic plasticizer”^43^. It is commonly used as a polyol in the preparation of hydrocolloid films. Glycerol is known to be effective in preventing brittleness in plasticized hydrophilic polymers^44^. Being a hydrophilic plasticizer, incorporation of glycerol increases the permeability and water sorption properties of edible / biodegradable polymers without affecting their mechanical tensile properties^45^. Due to growing health concerns and the potential negative environmental impact of leachable synthetic plasticizers, the use of “natural hydrophilic plasticizers” are now preferentially being used in the preparation of edible / biodegradable food packaging films.

PHB and melanin are both non-toxic biopolymers, which have a high plasticizing effect, while PHB is a well-known thermostable polymer that is biodegradable under both aerobic and anaerobic conditions^46^. Arreita and co-workers have previously incorporated limonene into a PHB-polylactic acid film polymer to provide a more flexible structure^26^. XRD analysis revealed that the nanocomposite film prepared here was crystalline in nature with results being very similar to the 2θ values reported in the literature except for a few reflections observed at 32.6°, 37.6° and 46.3°; showing that the blend was more highly crystalline in nature than the nanocomposite film that was previously prepared using thermo-compression molding^47^. When compared with the XRD analysis of PHB^48^ the 2θ values were different which appears to indicate that the Nm-PHB blend with glycerol may promote interactions between the molecules resulting in the additional peaks observed in the present study. The DSC results clearly demonstrated the thermostable nature of the nanocomposite film, which was up to 281.87 °C in the film prepared using the Nm-PHB blend with glycerol. This compares very favorably with the thermostability of PHB films prepared using acetic acid casting which displayed thermal stability up to 160 °C^25^.

Our study is the first to report on a PHB: Nm: glycerol polymer film that is odorless, flexible and nontoxic and which shows antimicrobial and antioxidant activities. Therefore the Nm-PHB nanocomposite film could be useful as packaging material for food products, where it could not only protect the food products from oxidation but also from the formation of potential biofilms by pathogenic bacteria. Melanin produced by the isolate WH001 55 was found to be soluble in various solvents including water, aqueous NaOH, and dimethyl sulfoxoide which suggests that it could also be used as a biomaterial in tissue engineering applications^49^. Melanin and PHB are known to be compatible biomaterials having previously been exploited in medical applications^14, 48, 50^.

Considering the antiadhesive effect of PHB^48^ and the bactericidal effect of Nm, we hypothesized a synergistic effect in the Nm-PHB film. In this study, the microtiter plate assay and CLSM image analysis showed that Nm PHB film was very effective against the formation of biofilm by *S*. *aureus*. MDR *S*. *aureus* is a highly prominent clinical pathogen, and is the causative agent of nosocomial infections and biofilm infections in indwelling medical devices. Once established these MDR *S*. *aureus* infections are largely recalcitrant to antibiotic treatment^51^ resulting in MDR *S*. *aureus* being a common biofilm associated infection in indwelling medical devices, ultimately leading in many cases to implant failure. Therefore, biomaterials with the ability to prevent/inhibit MDR *S*. *aureus* biofilms, such as the PHB: Nm: glycerol polymer film described here could also be very useful in combating such infections in medical implant devices. In addition the fact that the Nm-PHB nanocomposite film can be fabricated without the use of any organic solvents and chemicals increases the attractiveness of its potential use from an environmental perspective.

## Materials and Methods

### Isolation of sponge associated bacteria

The marine sponge *Tetyrina citrina* was collected by scuba –diving in early winter at Lough Hyne Marine Nature Reserve (N 51°30, W 9°18) at a depth of 15–20 m. To isolate bacteria 1 cm^3^ of sponge tissue was surface sterilized with sterile artificial seawater and the tissue was finely chopped and vortexed^52^. The sponge mixture was then serially diluted and plated on SYP-SW agar plates (Starch Yeast Peptone-Sea Water). The composition of SYP-SW media include (starch- 1%,yeast extract- 0.2%, peptone- 0.2%, artificial sea salts- instant ocean (aquatic ecosystems Inc., Apopka, FL, USA)-3.33%, agar-1.5%) and these plates were incubated at 28 °C for 5 days. Morphologically distinct bacteria were collected, stock cultures were prepared and stored at −80 °C.

### Screening and selection of melanin producing bacteria

A total of 66 distinct bacteria were isolated from the sponge *Tetyrina citirna*. These bacteria were screened for melanin production on tyrosine agar plates which were incubated at 28 °C for 6 days^33^. The plates were observed for the formation of zones of brown to black color pigments around the colonies. Melanin producing isolates were subsequently identified based on morphological and phylogenetic analysis.

### Production and extraction of melanin

The melanin producing isolate WH001 55 was inoculated into 100 ml of production media containing LB broth (Luria Bertani) supplemented with 1% tyrosine and incubated at 28 °C for 6 days at 180 rpm on an incubator shaker (Orbitek). The flasks were monitored for the production of a brown pigment and then samples were collected and centrifuged at 10,621 xg for 15 min. Samples were quantified for melanin yield and biomass was quantified by washing the precipitated pellet in phosphate buffered saline of pH 7.0 and allowed to air dry overnight. The melanin containing pellet was then acidified to pH 3 with 5 N HCl to ensure precipitation of the melanin. The precipitated melanin was centrifuged (Eppendorf) at 10,621 xg for 15 min, washed three times with deionized water, and lyophilized for dry weight determination.

### Media formulation and optimization of culture conditions to enhance melanin yield

Various media sources such as carbon, nitrogen and salt were investigated for enhanced production of melanin. The optimization of carbon sources was performed with supplementation of 1% each of glucose, sucrose, fructose, and starch in the production media. The optimization process was continued by testing various carbon sources in the 0.5 and 2.5%. range. The nitrogen sources including yeast extract, beef extract, peptone and ammonium nitrate were optimized under one-factor at a time experiments. The salt required for enhanced melanin yield was optimized using NaCl in the 0.5 to 2.5% range.

### Characterization of melanin

Lyophilized melanin was applied to silica gel TLC (Thin layer chromatography) plates and a chromatogram was developed using the solvent system of *n*-butanol: acetic acid: water (70:20:10). The developed plates were dried in an oven and the spots were visualized with ninhydrin. The TLC purified melanin was applied to a DEAE-Cellulose (Bio-Rad, 1 × 30 cm) (Diethylaminoethyl cellulose) column which had been equilibrated with 25 mM Tris–HCl buffer (pH 8.6) containing 50 mM sodium chloride. Elution was performed at a flow rate of 100 ml/h with 1:1 volume gradient from 0.1 M to 2 M NaCl in the same buffer^33^. The purified melanin was dissolved in deionised water and absorbance was recorded in the UV/VIS range at wavelengths between 200–450 nm in a spectrophotometer. Fourier transform infrared (FT-IR) spectral analysis was carried out in KBr disks on a Thermo-Nicolet Nexus 6700 FTIR spectrophotometer (Thermo Fisher Scientific Inc., Madison, WI, USA), and spectra were recorded in the wave number region of 400–4000 cm^−1^. The heat stability of the melanin was determined by increasing the temperature in a range from 40–100 °C and incubating for 10 min at the respective temperatures. The solubility of the melanin pigment was determined in deionized water, and in organic solvents such as ethanol, methanol, acetone, chloroform, hexane, ethyl acetate and benzene.

### Synthesis of nanomelanin (Nm)

The lyophilized melanin was dissolved in distilled water and sonicated at 30% amplitude, pulsing every 15 seconds for a total of 30 minutes (Millipore). The sonicated suspension was stirred vigorously for another 30 min and the size of the particles which were formed was analyzed using a UV spectrophotometer (PG Instruments), SEM (Carl Zeiss Evo 18) and particle size analysis performed using a laser diffraction particle size analyzer (Beckman-Coulter, LS-230, Miami, FL, USA). The particle size distribution (volume percent) was produced by the computer-controlled Coulter LS 230 instrument using the Beckman-Coulter LS 13 320 software program according to the principle of light scattering.

### Antimicrobial and antioxidant activity of Nm

The antimicrobial activity of Nm was determined using a well diffusion assay. *Staphylococcus aureus* MTCC 96, *E*. *coli* MTCC 41*, Pseudomonas aeruginosa* MTCC 3542 and *Bacillus cereus* MTCC 430 were grown in LB broth at 37 °C for 12 h. 100 µl of each respective bacterial culture was spread on the surface of the LB agar plate using sterile glass beads. Wells were made with a sterile steel cork borer and 20 µl of Nm suspension were added to the wells. The plates were incubated at 37 °C for 24 h and the wells were monitored for potential zones of inhibition. The bacterial growth inhibition assay was performed using a microtitre plate assay. Briefly, overnight cultures of each test strain was inoculated in 200 µl of LB broth in 96 well flat bottomed microtitre plates. Different concentration of 10–80 µg/ml of melanin and Nm was aliquoted in triplicate wells. Based on dose selecting experiments, 80 µg/ml of melanin and 30 µg/ml of Nm respectively were used in triplicate wells. Plates were incubated at 37 °C for 24 h. The absorbance was measured at 590 nm in a microtitre plate reader (Lanbics). Wells without melanin and Nm and LB broth acted as both positive and negative controls.

The antioxidant activity of Nm was determined using standard spectrophotometric based approaches^33, 53^. Briefly, the assay mixture was prepared in a screw cap tube using different concentration of melanin (0.5–1.5 mg/ml) dissolved in phosphate buffer of pH 7.0, mixed with 2 ml of reagent solution (0.6 M sulfuric acid, 28 mM sodium phosphate, and 4 mM ammonium molybdate) and incubated in a heating block at 95 °C. The absorbance of the mixture was recorded at 695 nm at regular intervals of 30 to 120 min. The DPPH (2, 2-diphenyl-1-picrylhydrazyl) assay was performed as previously described^54^. DPPH scavenging ability was calculated as [1 − (A_sample _− A_blank_) A_control_] × 100%.

### Fabrication of Nm-PHB nanocomposite film

To increase the thermal stability of the Nm film, a PHB polymer was blended and reinforced with Nm film. The PHB used was produced using the *B*. *casei* MSI04 strain supplemented with carbon source in minimal media as previously described^48^. Briefly, the PHB pellet was lyophilized and digested using 30% sodium hypochlorite followed by centrifugation at 10,621 xg and washing with distilled water, acetone, and ethanol. The final extraction was performed by adding boiling chloroform and H_2_SO_4_. The slurry was evaporated to dryness to obtain the PHB. Film fabrication was performed in three different combinations. The first combination included 1% (w/v) of PHB and 1% (w/v) of Nm blended with 1% glycerol and mixed using constant agitation and heated at 50 °C for 30 min. The PHB-Nm -glycerol mixture was subjected to homogenization to produce fine solutions. The solutions thus obtained after homogenization were sonicated for about 1 h at 50 kHZ to remove any air bubbles. 15 ml of this aliquot was spread on a petri dish and allowed to dry in a hot air oven at 60 °C for 36 h. Similarly two other combinations used for film preparation included increased concentrations of Nm blend (2%) and equal proportion of glycerol and PHB (1%). An alternative combination included increased PHB (2%) and 1% each of the Nm blend and glycerol.

### Characterization of Nm-PHB nanocomposite film

#### Surface imaging of films

The surface morphology of the thin films was assessed using scanning electron microscopy (FEI Quanta FEG 200-High Resolution SEM). Briefly, the developed film was deposited on aluminum stubs and coated with a gold layer using a sputtering coater with an accelerating voltage of 10–15 kV. All the prepared films of different combinations were scanned and the film morphology, and characteristics were determined.

#### XRD and AFM analysis

The degree of crystallinity of the film prepared using the three different combinations was estimated using XRD (X - ray diffraction) patterns obtained using a Rigaku (30 kv/25 mA) Geigerflex D/Mac, C series diffractometer (Tokyo, Japan) with Cu-kα radiation (λ = 1.5406 A°) at room temperature in glancing inclined angle mode. XRD analysis of the film was carried out as described in ref. 25 with necessary modifications. Briefly, the film was placed on a glass slide and baseline corrections were carried out, then hot pressed at 100° for 2 min to erase the residual thermal history, and subsequently quenched to room temperature, followed by maintenance for 24 h to allow complete crystallization. The scanning range was 10–0° at a rate of 0.02°/second. Based on SEM and XRD analysis effective film prepared using 1% each of Nm, PHB, and glycerol blend was determined and the AFM ((Atomic Force Microscopy) analysis was carried out to determine the film roughness and surface morphology. The route mean squared (RMS) roughness values were estimated with the help of the NanoScope analysis software. AFM (diCaliber AFM, Bruker), was operated in the tapping mode with a spring constant of 40 N/m^25^.

### Differential scanning calorimetry (DSC) of the film

The film combinations of 1% each of (PHB, Nm, glycerol) were selected based on SEM and XRD analysis. The thermal property of the film was determined in a differential scanning calorimeter (DSC Q20 V24.2 Build 107). Briefly DSC was performed by placing the film in aluminum pans over the temperature range from 0 °C to 400 °C at a heating rate of 10 °C/min. The flow rate of the gas was 50 ml/min. The weight loss was recorded as a function of temperature. The heating rate and gas flow was maintained as uniform throughout the film and the control (melanin).

### Protective effect of Nm-PHB film against MDRSA

In preliminary experiments, a number of clinical isolates of multiple drug resistant *S*. *aureus* (MRDA) were rescreened on antibiotics such as kanamycin, amoxicillin, ampicillin and oxacillin (Himedia), to confirm their phenotype. A *S*. *aureus* strain which displayed resistance to all the antibiotics was confirmed as a MDR *S*. *aureus* and was subsequently used in the biofilm assay. Microtitre biofilm assays were performed as previously described with a few modifications^55^. Briefly, a culture of *S*. *aureus* was grown in nutrient rich LB medium. The overnight grown culture was diluted to 1:100 and inoculated into fresh LB medium. The assay was performed in 12 deep polystyrene well plates (sigma) in triplicate. Tests wells were coated with a) varying concentration of Nm 10–30 µg/ml and b) Nm:PHB composite. Uncoated wells with *S*. *aureus* culture were used as the positive control and Nm:PHB composite coated wells without *S*. *aureus* culture was used as the negative control. The *S*. *aureus* culture (100 µl containing ~10^4^ cells/ml) was inoculated into all the wells and the plates were incubated at 37 °C for 48 h. After incubation, the planktonic cells were removed by inverting the plate over sterile paper towels. The plates were submerged in water to remove the unattached cells and media components. The wells were then stained with 125 μl of 0.1% crystal violet solution, and incubated for 10–15 min at 37 °C; followed by rinsing with water to remove excessive stain. The wells were imaged using a Nikon DSLR 5500 digital camera. After imaging, the wells were treated with 125 μl of 30% acetic acid in water to perform a quantitative estimation of biofilm formation in a microplate reader (Labnics) at 550 nm. A glass slide / film surface biofilm assay was performed to confirm antibiofilm activity using CLSM images. A broth culture of MDR *S*. *aureus* was allowed to form a biofilm on the nanocomposite Nm-PHB film surface at 37 °C for 168 h. Control surface were set as a glass slide and a µ slide. Antibiofilm assays were performed in triplicates. The Nm-PHB film and control surfaces were washed with PBS, dried and stained with 0.1% acridine orange. Excess stain was washed, air-dried and the biofilm formed was visualized under CLSM (Carl Zeiss LSM 710)^56^.

## Electronic supplementary material

Supplementary information.
