# Supplementary material for: Synthesis of Nm-PHB (nanomelanin-polyhydroxy butyrate) nanocomposite film and its protective effect against biofilm-forming multi drug resistant Staphylococcus aureus
Source: Sci Rep. 2017 Aug 22;7:9167. doi: 10.1038/s41598-017-08816-y (PMC5567312; doi:10.1038/s41598-017-08816-y)
Supplement: Supplementary file 1 — Supplementary information. [file 41598_2017_8816_MOESM1_ESM.doc]

**Synthesis of Nm-PHB (nanomelanin-polyhydroxy butyrate) nanocomposite film and its protective effect against biofilm-forming multi drug resistant *Staphylococcus aureus***

George Seghal Kiran^1*^, Stephen A Jackson^2^, Sethu Priyadharsini ^1^, Alan D.W. Dobson^2^, Joseph Selvin^3^


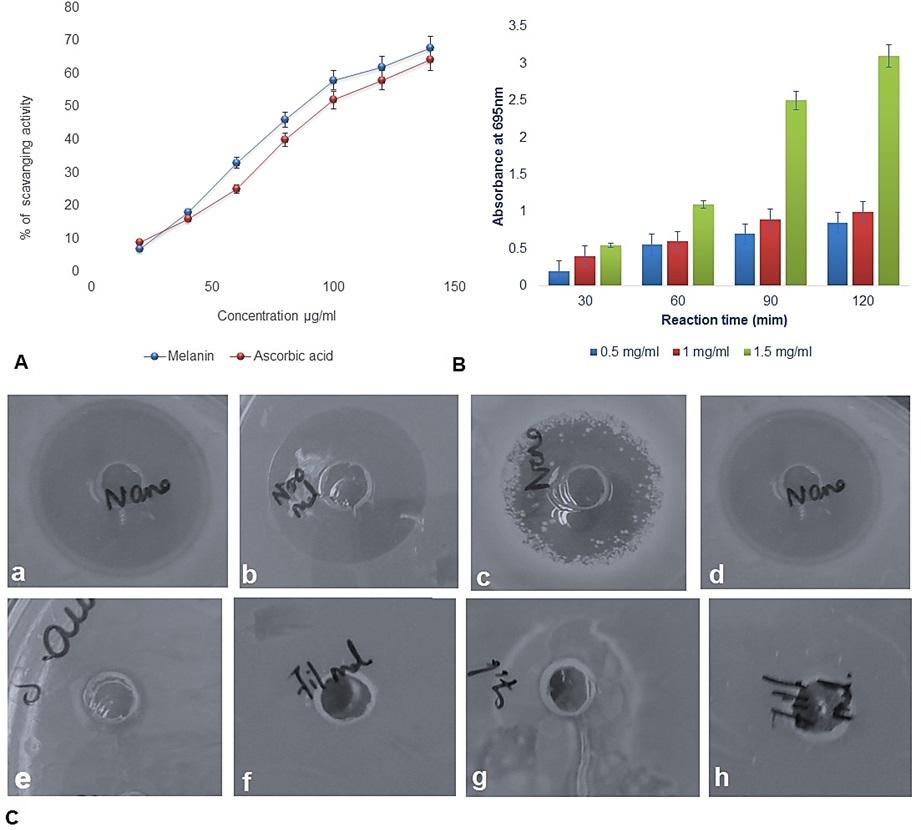


**Supplementary Figure** S1. **A)** Dose dependent scavenging activity of melanin as evident from the DPPH assay using ascorbic acid as the control. **B**) Spectrophotometric reduction assay of melanin

**A**
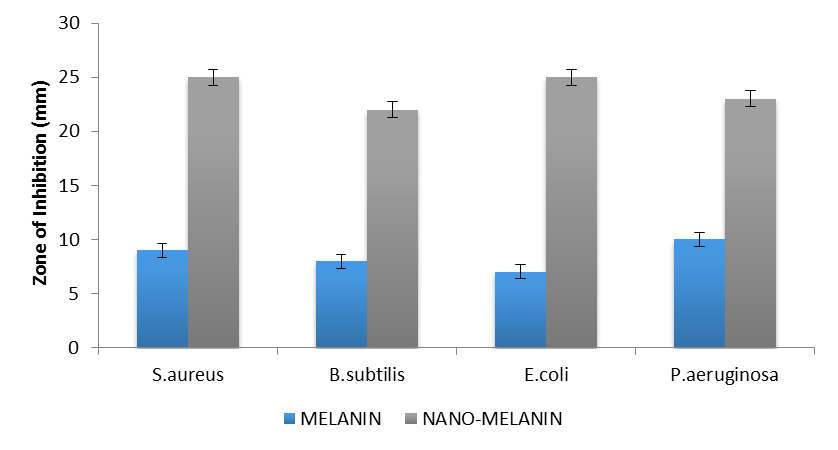


**B**
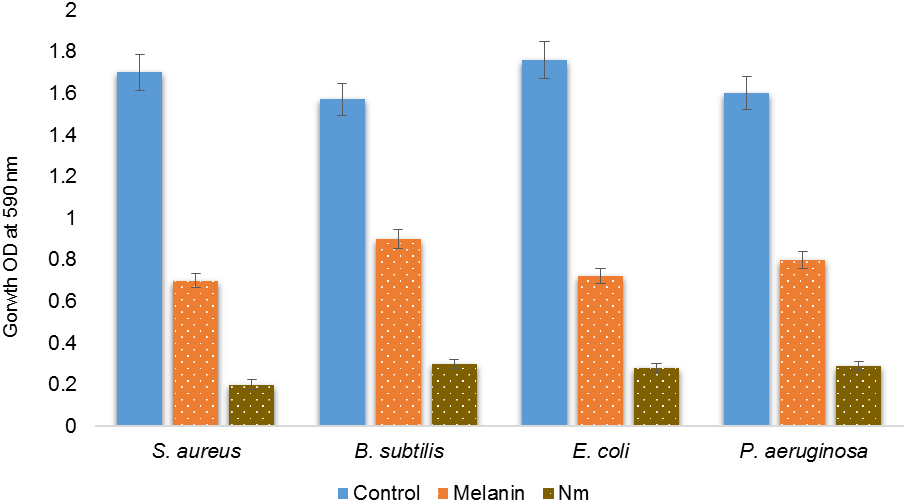


**Supplementary Figure S2**. **A**) Antimicrobial activity of Nm against a. *S. aureus*, b. *E.coli*, c. *Pseudomonas aeruginosa* d. *Bacillus subtilis* and e- h. antimicrobial activity of melanin against *S. aureus*, *E. coli*, *Pseudomonas aeruginosa* and *Bacillus subtilis*. **B)** Antibacterial activity (performed in microtitre plate assay) of melanin and Nm on the growth of pathogens. Untreated bacterial culture was set as control. Comparatively Nm has better inhibition than melanin. The error bars are measure of standard error (n = 3).

Determination of Mechanism of action of Nm

The ration of MBC:MIC was used to classify the mode of action of Nm on pathogenic bacteria tested. The test and classification of activity was performed as per Clinical and Laboratory Standard Institute (CLSI) guidelines (2009). As per the activity classification, the ratio of MBC:MIC is <4.0, the Nm concentration was noted as bactericidal. 100 µl of MIC well and the 2 dilutions above MIC were spread on LB agar and incubated as 37°C for 24 h. The MBC was noted at the lowest concentration showed no growth of colonies on the plate in comparison to other concentrations and control.

**Results**

Table 1. Determination of effective concentration of Nm against Gram positive and Gram negative pathogens tested

| Nm concentration (µg/ml) | SA | EC | PA | BS |
| --- | --- | --- | --- | --- |
| 18 | 0.7±0.01 | 0.72±0.02 | 0.78±0.02 | 0.8±0.03 |
| 22 | 0.42±0.01 | 0.4±0.03 | 0.46±0.01 | 0.5±0.01 |
| 26 | 0.2±0.03 | 0.3±0.01 | 0.24±0.02 | 0.31±0.01 |
| 30 | 0.2±0.02 | 0.26±0.01 | 0.2±0.02 | 0.28±0.015 |
| 34 | 0.2±0.01 | 0.2±0.011 | 0.2±0.01 | 0.24±0.021 |
| 38 | 0.2±0.01 | 0.2±0.02 | 0.2±0.012 | 0.2±0.01 |
| C | 1.72±0.01 | 1.76±0.01 | 1.6±0.02 | 1.58±0.01 |

n = 3 (triplicates). SA - *S. aureus*, EC – *E. coli*, PA – *P. aeruginosa*, BS – *B. subtilis*

Among the tested concentrations, 30 µg/ml and above was considered as effective concentration which showed lowest growth OD corresponding to the effective concentration of Nm (Table S1). Based on the MBC:MIC ratio, the mode of action of Nm was classified as “bactericidal” (Table S2). The antimicrobial activity was effected on both Gram negative and Gram positive bacteria and therefore Nm was grouped under broad-spectrum antibacterial compound.

[Table](http://aac.asm.org/content/53/5/1735.full#sec-1) S2. MIC, MBC and MBC:MIC ratio of Nm

|  | SA | EC | PA | BS |
| --- | --- | --- | --- | --- |
| MIC (µg/ml) | 28±1.4 | 36±2.6 | 32±1.2 | 38±0.6 |
| MBC (µg/ml) | 32±0.4 | 42±0.7 | 34±0.6 | 42±1.2 |
| MIC/MBC | 0.87 | 0.85 | 1.06 | 0.9 |

SA - *S. aureus*, EC – *E. coli*, PA – *P. aeruginosa*, BS – *B. subtilis*

***Antibacterial effect of Nm-PHB film***

The antibacterial assay plates were prepared with *S. aureus* lawn on LB agar plates and Nm-PHB film (5, 10 and 25 mm diameter) was placed over lawn. The plates were incubated at 37ºC for 24 h and were observed for zones of inhibition. The inhibition zone was insignificant and was ≤ 1 mm in all test plates include 5, 10 and 25 mm dia Nm-PHB film. The bactericidal effect of Nm was not revealed in the Nm-PHB film may be due to lowest diffusion of Nm in the media from Nm-PHB film. Therefore, the antibiofilm assays were performed based on the hypothesis that the film might have a synergistic effect (PHB showed antiadhesive activity and Nm showed bactericidal activity).

References

 Clinical and Laboratory Standard Institute (CLSI). Methods for dilution antimicrobial susceptibility tests for bacteria that grow aerobically; approved standard, CLSI document M07-A8. 8th. Wayne, Pennsylvania: Clinical and Laboratory Standards Institute; 2009.
